# Genome-Wide Association Analysis of Imputed Rare Variants: Application to Seven Common Complex Diseases

**DOI:** 10.1002/gepi.21675

**Published:** 2012-09-05

**Authors:** Reedik Mägi, Jennifer L Asimit, Aaron G Day-Williams, Eleftheria Zeggini, Andrew P Morris

**Affiliations:** 1 Estonian Genome Centre, University of TartuTartu, Estonia; 2 Wellcome Trust Sanger InstituteHinxton, United Kingdom; 3 Wellcome Trust Centre for Human Genetics, University of OxfordOxford, United Kingdom

**Keywords:** genome-wide association study, rare variants, imputation

## Abstract

Genome-wide association studies have been successful in identifying loci contributing effects to a range of complex human traits. The majority of reproducible associations within these loci are with common variants, each of modest effect, which together explain only a small proportion of heritability. It has been suggested that much of the unexplained genetic component of complex traits can thus be attributed to rare variation. However, genome-wide association study genotyping chips have been designed primarily to capture common variation, and thus are underpowered to detect the effects of rare variants. Nevertheless, we demonstrate here, by simulation, that imputation from an existing scaffold of genome-wide genotype data up to high-density reference panels has the potential to identify rare variant associations with complex traits, without the need for costly re-sequencing experiments. By application of this approach to genome-wide association studies of seven common complex diseases, imputed up to publicly available reference panels, we identify genome-wide significant evidence of rare variant association in *PRDM10* with coronary artery disease and multiple genes in the major histocompatibility complex (MHC) with type 1 diabetes. The results of our analyses highlight that genome-wide association studies have the potential to offer an exciting opportunity for gene discovery through association with rare variants, conceivably leading to substantial advancements in our understanding of the genetic architecture underlying complex human traits.

## INTRODUCTION

There has been much recent debate as to the role of rare genetic variation, defined here to have a minor allele frequency (MAF) of less than 1%, in explaining the ‘missing heritability’ of complex traits [Dickson et al., 2010; Frazer et al., 2009; Yang et al., 2010. Rare variants are likely to have originated from founder effects in the last 20 generations, and thus are more likely to be population specific [Bodmer and Bonilla, 2008. They are also likely to have larger effects on complex traits than common variants, consistent with the expectation that they will have been subject to purifying selection after recent expansion of the human population [Pritchard, 2001. However, these effects are unlikely to be sufficiently large to be detected through association testing with individual rare variants. Statistical methods have thus focussed on the aggregation of the effects of all rare variants within the same exon, gene or pathway, potentially weighting according to annotation or MAF [Han and Pan, 2010; Hoffman et al., 2010; Li et al., 2010; Li and Leal, 2008; Madsen and Browning, 2009; Morgenthaler and Thilly, 2007; Morris and Zeggini, 2010; Neale et al., 2011; Price et al., 2010; Wu et al., 2011; Zawistowski et al., 2010]. Using these methods, multiple rare variants have been demonstrated to be associated with a variety of complex traits including low- and high-density lipoprotein [Cohen et al., 2006; Romeo et al., 2007, body mass index [Ahituv et al., 2007 and blood pressure [Ji et al., 2008].

The most comprehensive approach to characterising the contribution of rare variants to the genetic component of complex traits is through large-scale, next-generation re-sequencing studies [Metzker, 2010]. Despite improvements in the throughput and efficiency of these technologies, rare variant re-sequencing efforts on the scale of the whole genome still represent an infeasible financial undertaking for most research groups. Consequently, most rare variant studies have focussed on candidate genes, or more recently, the exome [Ng et al., 2010]. However, high-density reference panels obtained from whole-genome re-sequencing data are being released through the 1000 Genomes Project, providing a comprehensive catalogue of variation with MAF as low as 0.5%, as well as many rarer variants, across a wide range of populations from different ethnic groups [The 1000 Genomes Project Consortium, 2010]. Such reference panels could be utilised to select rare variants for large-scale genotyping with custom designed arrays, potentially with priority given to variants with likely functional consequences in an effort to reduce costs, such as the Illumina Infinium HumanExome BeadChip. Conversely, genome-wide association study (GWAS) genotyping products have been designed primarily to capture common genetic variation, and thus offer poor coverage of rare variants [Barrett and Cardon, 2006]. However, if samples have already been assayed by means of such a GWAS chip, imputation techniques [Marchini and Howie, 2010] can make use of this existing scaffold to predict genotypes at variants present on the higher density reference panel, incurring no additional cost, other than computation, although this is far from trivial.

Here, we formulate methodology for the detection of complex trait association with accumulations of minor alleles within genes, or some other functional unit, using data from directly typed and/or imputed rare variants. We report the results of simulations to investigate the power of alternative design strategies for assaying and characterising rare genetic variation to detect association with a quantitative trait in a 50 kb gene. Our study considers a simple model of rare variant association with the trait, and assesses the impact on power of the number of individuals present in the reference panel. We also present results of an application of our methodology to rare variant association analysis of seven common complex diseases undertaken by the Wellcome Trust Case Control Consortium (WTCCC) [The Wellcome Trust Case Control Consortium, 2007], using GWAS data imputed up to the Phase I 1000 Genomes Project reference panel (June 2011 interim release) [The 1000 Genomes Project Consortium, 2010].

## METHODS

### MODEL FORMULATION

We test for association of a complex trait with accumulations of minor alleles at *N* rare variants, here defined to have MAF less than 1%, within the same exon, gene or some other functional unit, in a sample of unrelated individuals. Let *n_i_* denote the number of rare variants at which the *i*th individual has been successfully genotyped in the functional unit. Furthermore, let *G_ij_* denote the genotype of this individual at the *j*th rare variant, coded as 0 for the common homozygote, and 1 otherwise. We can model the phenotype, *y_i_*, of this individual in a generalised linear regression framework as a function of the proportion of rare variants at which they carry at least one minor allele [Morris and Zeggini, 2010], given by

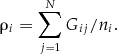

Specifically, 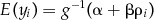, where *g* is the link function and *β* is the expected increase in the phenotype for an individual carrying a full complement of minor alleles at rare variants in the functional unit compared to an individual carry none. It follows that *β*/*N* is the increase in the expected phenotype of an individual for each rare variant at which they carry a minor allele. The likelihood contribution of the *i*th individual, 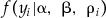, is weighted by *n_i_* to allow for differential call rates between samples. We can thus construct a likelihood ratio test by comparing the maximised weighted likelihoods of two models via analysis of deviance: (i) the null model where *β* = 0; and (ii) the alternative model for which *β* is unconstrained. The resulting test statistic has an approximate *χ*^2^ distribution with one degree of freedom. The flexibility of the generalised linear regression framework allows for generalisation of this approach to take account of non-genetic risk factors as covariates.

It is straightforward to accommodate imputed rare variants in the functional unit within the generalised linear regression framework by considering the posterior distribution of genotype calls. Specifically, we replace missing genotypes (at typed variants, or at untyped variants in the imputation reference panel) by their expectation, *E*(*G_ij_*) = 1 – *h_ij_*, where *h_ij_* is the posterior probability of a common homozygote call in the *i*th individual at the *j*th rare variant. The posterior probabilities, *h_ij_*, of imputed genotype calls are easily recovered from standard imputation software such as IMPUTEv2 [Howie et al., 2009 and BEAGLE [Browning and Browning, 2009.

The methodology described above has been implemented in the GRANVIL software, and is freely available for download (http://www.well.ox.ac.uk/GRANVIL). The open-source software has been designed to efficiently handle analysis of directly genotyped and imputed rare variants on a genome-wide scale, and can accommodate both quantitative traits and binary phenotypes in a generalised linear modelling framework, as described above. The user supplies a file containing the boundaries of each functional unit to be analysed, together with SNPTEST format sample and genotype files, and specifies the rare variant MAF threshold. GRANVIL is distributed with scripts to generate graphical summaries of GWAS rare variant analysis results, including quantile-quantile and Manhattan plots.

### SIMULATION STUDY

We have performed simulations to evaluate the relative performance of different design strategies to identify quantitative trait association with rare variants in a 50 kb gene. We have considered an analysis cohort of 2,000 individuals, and a reference panel ascertained from the same population. We have compared the power of GRANVIL in the following scenarios: (i) direct re-sequencing of the analysis cohort; (ii) direct genotyping of the analysis cohort for all rare variants present in the reference panel; (iii) direct genotyping of the analysis cohort for variants on a GWAS chip with the same characteristics as the Illumina Human660W-Quad BeadArray in terms of density and MAF; and (iv) direct genotyping of the analysis cohort for variants on the GWAS chip, supplemented with imputation of untyped variants present in the reference panel using IMPUTEv2 [Howie et al., 2009].

We have considered a simple underlying model for the association of the trait with multiple rare causal variants within the same gene. We assumed that the expected trait value of each individual is increased by the presence of a minor allele at any causal variant. The trait association model was then parameterised in terms of: (i) the maximum MAF, *δ*, of any individual causal variant; (ii) the total MAF, *Q*, of all causal variants in the region; and (iii) their joint contribution to the overall trait variance, expressed as 100*λ*%. Here, we considered reference panels of *R* = 120, *R* = 500 and *R* = 4,000 individuals. The number of individuals was chosen to represent a range of reference panels incorporating those available from the 1000 Genomes Project (pilot release), through to those we might expect from future large-scale deep re-sequencing efforts, such as the UK10K initiative (http://www.uk10k.org/). For each model, we generated 500 replicates of data as follows.

1. Generate an ancestral recombination graph [Griffiths and Marjoram, 2007] for a population of 40,000 haplotypes from a realisation of the coalescent process with recombination, obtained using the MS software [Hudson, 2002]. We assumed a mutation rate of 10^−8^ per base (in each generation) and a uniform recombination rate of 1 cM per Mb, for an effective population size of 10,000 individuals. In total, we simulated a region of 1,050 kb, including a 50 kb gene and 500 kb up- and down-stream to allow for an imputation buffer to improve accuracy by avoiding edge effects and taking advantage of the expected long-range linkage disequilibrium (LD) with rare variants [The International HapMap Consortium, 2007.
2. Calculate the MAF at each variant across the 50 kb gene in the population of 40,000 chromosomes, denoted by *q_j_* for the *j*th variant. Select a variant as causal from amongst those with MAF *q_j_* < *δ*, at random. Continue selecting causal variants in this way, without replacement, until the total MAF of all causal variants is *Q*.
3. Select a random sample of 4,000 chromosomes from the population, paired together to form the analysis cohort. Determine the number of minor alleles carried by the *i*th individual across all causal variants in the 50 kb gene, denoted by *m_i_*. The phenotype, *y_i_*, of the *i*th individual is then simulated from a Gaussian *N*(*μ_i_*,*σ*) distribution, where *σ* is determined by the spectrum of causal variants and their joint contribution, *λ*, to the overall trait variance, and *μ_i_* = 1 if *m_i_* > 0, and 0 otherwise. Full details of the derivation of the residual trait variance, *σ*^2^, are provided in the Appendix.
4. Select a random sample of 2*R* chromosomes from the remainder of the population to be haplotypes in the reference panel. Assuming no genotyping or phasing errors in the reference panel, record the haplotype of each of these chromosomes across all variants in the 1,050 kb region.
5. Begin by considering the strategy in which the analysis cohort has been directly re-sequenced in the 50 kb gene. Assuming no sequencing errors, record the genotype of each individual at each variant with MAF < 1% in the analysis cohort. Test for association of the quantitative trait with an accumulation of minor alleles at these variants using GRANVIL, and record the *P*-value, denoted by *p_SEQ_*.
6. Then consider the scenario in which the analysis cohort has been directly genotyped for all variants in the 50 kb gene which are present in the reference panel. Assuming no genotyping errors, record the genotype of each individual at each variant present in the reference panel with MAF < 1% in the analysis cohort. Test for association of the quantitative trait with an accumulation of minor alleles at these variants using GRANVIL, and record the *P*-value, denoted by *p_GEN_*.
7. Next consider the scenario in which the analysis cohort has been directly genotyped only for variants on a GWAS chip. Select a random 1,050 kb region of the genome, and determine the number of variants, *n_GWAS_*, present on the Illumina Human660W-Quad BeadArray in that interval. Select *n_GWAS_* variants at random and without replacement, with ascertainment probability 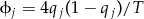, as present on the chip, where 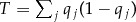. This probability density incorporates the strong bias towards common variants on GWAS chips, generating an approximately uniform distribution of MAF [Anderson et al., 2008. Assuming no genotyping errors, record the genotype of each individual at each variant within the 50 kb gene with MAF < 1% in the analysis cohort. Test for association of the quantitative trait with an accumulation of minor alleles at these variants using GRANVIL, and record the *P*-value, denoted by *p_GWAS_*.
8. Finally, consider the scenario in which the analysis cohort has been directly genotyped only for variants on the GWAS chip, but are subsequently supplemented with imputation of untyped variants present in the reference panel. Assuming no genotyping errors, record the genotype of each individual at each variant across the 1,050 kb region, irrespective of MAF. Impute the genotype of each individual in the analysis cohort at each variant present in the reference panel in the 50 kb gene using IMPUTEv2 [Howie et al., 2009, assuming an effective population size of 10,000 individuals and a buffer region of 500 kb. Test for association of the quantitative trait with an accumulation of minor alleles at directly genotyped and imputed variants within the 50 kb gene with MAF < 1% in the analysis cohort and ‘info score’ greater than 0.4 using GRANVIL, and record the *P*-value, denoted by *p_IMP_*.

Over all simulated data sets, we calculated the power of each design strategy at a nominal 5% significance level as the proportion of replicates for which the corresponding *P*-value is less than 0.05. We also calculated the mean numbers of rare variants in the 50 kb gene: (i) in the population of 40,000 chromosomes; (ii) identified through direct re-sequencing of the analysis cohort; (iii) present on the reference panel and identified through direct genotyping of the analysis cohort; (iv) present on the GWAS chip; (v) present on the reference panel and well imputed (info score greater than 0.4) in the analysis cohort.

One potential limitation of our simulation study is the assumption of no sequencing errors, perfect phasing of the reference panel, and no missing or miscalled genotypes. These errors might be expected to be most detrimental to our proposed imputation strategy since this process requires an accurate GWAS scaffold and phased reference panel. To address this issue, we have also performed simulations to assess the robustness of our results to re-sequencing errors and missing or miscalled genotypes. We introduced a simple model of errors [Hao et al., 2004 by randomly swapping the base call (ancestral or mutant) of each individual at each variant with probability ε. Here, we considered ε = 0% (no errors), ε = 0.1% (error rate of ∼0.2% in genotype calls) and ε = 0.2% (error rate of ∼0.4% in genotype calls). We evaluated the impact of call rate by removing genotypes, at random, with probability *κ*, where here we considered *κ* = 0% (no missing genotype data) and *κ* = 1%. Errors were introduced first into the reference panel (Step 4), and then into the analysis cohort (Steps 5, 6, 7 and 8). Missing genotype data were then introduced at random (Steps 6, 7 and 8).

### APPLICATION TO IMPUTED RARE VARIANT GWAS OF SEVEN COMMON COMPLEX DISEASES

We considered 14,000 cases of seven common complex diseases (bipolar disorder, coronary artery disease, Crohn's disease, hypertension, rheumatoid arthritis, type 1 diabetes and type 2 diabetes) and 3,000 shared controls from the WTCCC [The Wellcome Trust Case Control Consortium, 2007. Samples were ascertained from the United Kingdom and genotyped using the Affymetrix GeneChip 500K Mapping Array Set, which incorporates 500,568 single nucleotide polymorphisms (SNPs) genome-wide. We utilised the same quality control (QC) filters employed by the WTCCC to exclude samples and SNPs from the analysis, full details of which are presented in the description of the experiment [The Wellcome Trust Case Control Consortium, 2007. In brief, samples were excluded on the basis of low call rate, outlying genome-wide heterozygosity, discrepancies in WTCCC and external identifying information, non-European ancestry, duplication and apparent relatedness. SNPs were excluded on the basis of low call rate, extreme deviation from Hardy-Weinberg equilibrium (HWE), differential allele or genotype frequencies between the two control cohorts and manual visual inspection of genotype calls in cluster plots.

To allow for fine-scale population structure, which may have greater impact on rare variant association signals than common SNPs because of recent founder effects [Bodmer and Bonilla, 2008, we constructed principal components to represent axes of genetic variation within the UK. We applied EIGENSTRAT [Price et al., 2006 to a subset of high-quality LD-pruned SNPs (*r*^2^ < 0.2) with MAF of at least 5%, and projected samples onto principal components demonstrating clear separation between 12 UK regions of residence [The Wellcome Trust Case Control Consortium, 2007.

We imputed the high-quality samples up to the Phase I 1000 Genomes Project reference panel (June 2011 interim release) consisting of 1,094 phased individuals from multiple ancestry groups [The 1000 Genomes Project Consortium, 2010. We removed SNPs with MAF < 1% from the GWAS scaffold, prior to imputation, since we expect these variants are likely to be subject to higher genotyping errors, which may impact the downstream analysis. We performed imputation using IMPUTEv2 [Howie et al., 2009 with default parameter settings and sample pre-phasing, allowing a buffer region of 500 kb. Subsequently, we tested for association of each disease with ‘high-quality’ rare variants (MAF less than 1%, and IMPUTEv2 info score greater than 0.4) within genes using GRANVIL, adjusting for principal components as covariates to account for fine-scale UK population structure. Gene boundaries were defined from the UCSC human genome database (build 37).

## RESULTS

### SIMULATION STUDY

**Fig. 1:**
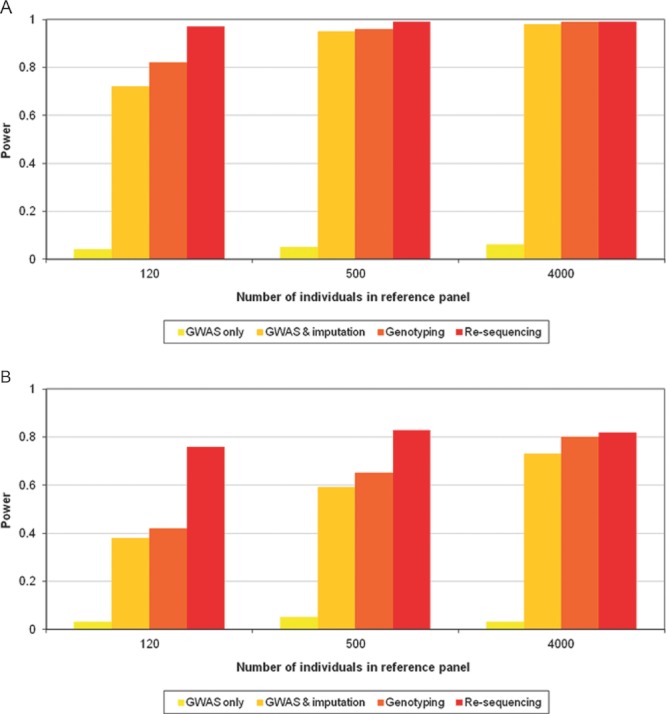
Power, at a nominal significance level of *P* < 0.05, to detect association of an accumulation of minor alleles with a quantitative trait, for different strategies for assaying rare genetic variation in a 50 kb gene, as a function of the size of the reference panel. Multiple causal variants in the gene contribute jointly to 5% of the overall trait variation. The panels correspond to two specific trait association models: (A) the maximum MAF of any individual causal variant is 1%, and the total MAF of all causal variants is 5%; and (B) the maximum MAF of any individual causal variant is 0.5%, and the total MAF of all causal variants is 2%.

Figure 1 presents the power, at a nominal significance level of *P* < 0.05, to detect association with a quantitative trait, for each of the design strategies for assaying rare genetic variation in the gene. For these results, we assumed that multiple rare causal variants in the gene jointly contribute to 5% of the overall trait variation. The panels correspond to two specific trait association models: (A) the maximum MAF of any individual causal variant is 1%, and the total MAF of all causal variants is 5%; and (B) the maximum MAF of any individual causal variant is 0.5%, and the total MAF of all causal variants is 2%. Under the second of these models, we expect fewer rare variants within the gene to be causal, since the total MAF is lower. A higher proportion of non-causal variants within the gene would be expected to reduce power overall, irrespective of the design strategy and/or the number of individuals in the reference panel [Morris and Zeggini, 2010.

Our results highlight a number of general conclusions across these trait association models. As expected, the most powerful strategy to detect rare variant association is to re-sequence the analysis cohort. In the absence of sequencing errors, this ‘gold-standard’ strategy provides complete coverage of rare genetic variation in the gene within the analysis cohort. Nevertheless, genotyping the analysis cohort for all rare variants present in the reference panel generally results in a relatively small reduction of power, particularly for *R* = 4,000. We expect most of the rare variation in the analysis cohort to be captured by such large reference panels (Supporting Information Figure S1). Rare variants not captured by the reference panel (e.g., private mutations) are less likely to have a major impact on the joint contribution of causal variation in the gene under our simulation model, and thus would not be expected to lead to a dramatic reduction in power.

As previously reported [Morris and Zeggini, 2010, genotyping of the analysis cohort with the GWAS chip alone has minimal power to detect association because very few rare variants within the gene are assayed directly (Supporting Information Figure S1). However, imputation into this GWAS scaffold in the analysis cohort up to the density of the reference panel can lead to substantial gains in power to detect rare variant association within the gene. The extent of the increase in power depends crucially on the number of individuals in the reference panel, although the gains from *R* = 500 to *R* = 4,000 are not as great as from *R* = 120 to *R* = 500, particularly for an association model incorporating causal variants with MAF up to 1% (Figure 1). Reference panels with more individuals provide more comprehensive coverage of rare variation in the region (Supporting Information Figure S1), higher quality imputation, and thus greater improvements in power. Note that the relative power of imputation appears lower under trait association model (A), where the maximum MAF of any causal variant is lower than under model (B). This is not unexpected since the distribution of causal allele frequencies will be more skewed to the rarest variants under this model, which we anticipate to be most difficult to impute, irrespective of the size of the reference panel.

We also considered the impact of sequencing errors and missing and/or miscalled genotypes on the power of the four alternative strategies. As expected, the power of all strategies is decreased as the error rates and the frequency of missing genotypes increase (Supporting Information Figure S2). However, we are still able to recover much of the power of the gold-standard re-sequencing strategy through imputation of the analysis cohort from the GWAS scaffold, and we maintain considerable advantages over genotyping of the GWAS chip alone.

### APPLICATION TO IMPUTED RARE VARIANT GWAS OF SEVEN COMMON COMPLEX DISEASES

**Fig. 2:**
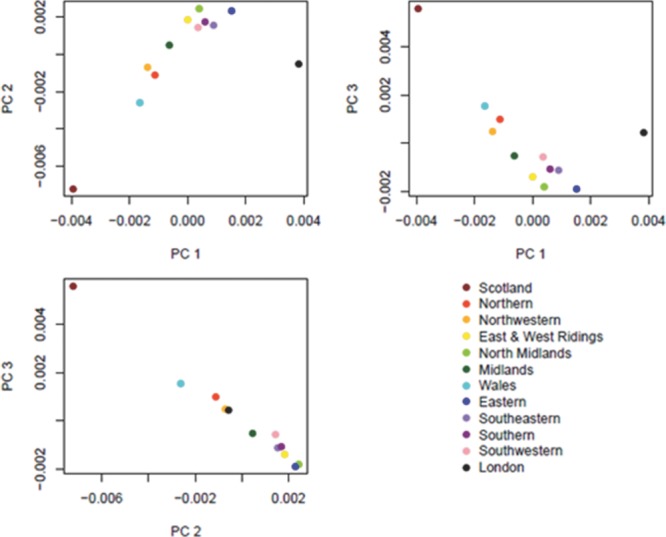
Principal components representing axes of genetic variation demonstrating clear separation between 12 UK regions of residence. Each point represents the mean projection of samples from each UK region onto the first three axes of genetic variation.

A total of 13,241 cases and 2,938 controls from the WTCCC experiment passed sample QC filters (Supporting Information Table S1). Of the autosomal variants on the array, 456,868 passed SNP QC filters. We then applied EIGENSTRAT to an LD-pruned (*r*^2^ > 0.2) set of 27,770 high-quality autosomal SNPs with MAF > 5% to construct 10 axes of genetic variation of UK population structure. By projecting samples onto the corresponding principal components, we observed that the first three axes of genetic variation were strongly associated with the region of residence of samples (Figure 2). The first principal component separated London and Scotland from the remainder of the United Kingdom, whilst the second and third principal components separated regions within the United Kingdom on a North-West to South-East axis. These three principal components were thus selected for adjustment of downstream association analyses to allow for fine-scale UK population structure.

After removal of variants with MAF < 1%, a total of 391,060 high-quality SNPs remained in the GWAS scaffold. A total of 8,239,134 rare variants were successfully imputed up to the Phase I 1000 Genomes Project reference panel (June 2011 interim release) [The 1000 Genomes Project Consortium, 2010 and were polymorphic in the WTCCC experiment. Of these, 5,383,228 (65.3%) had IMPUTEv2 info score of at least 0.4. Amongst these ‘well-imputed’ rare variants, the mean info score was 0.618, and 17.3% had info score greater than 0.8.

**Fig. 3:**
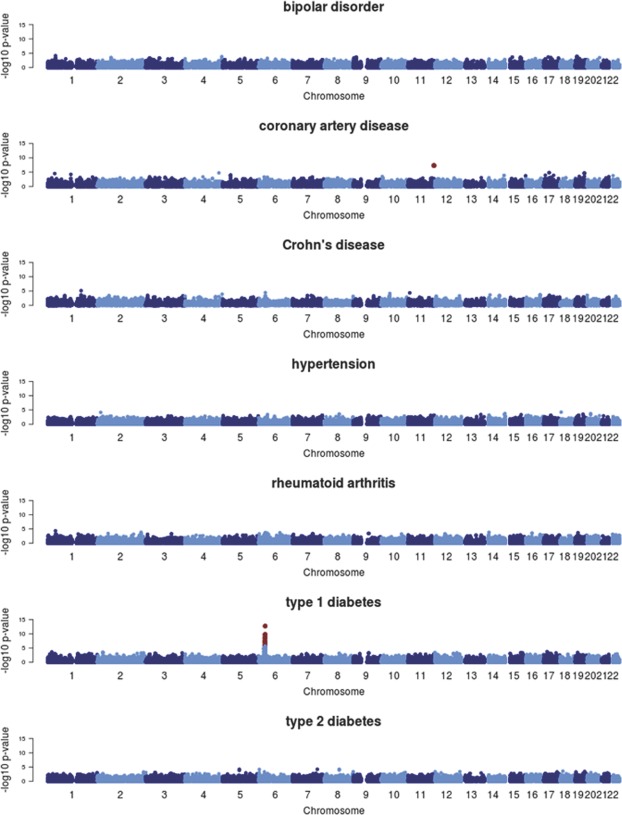
Manhattan plots summarising association of seven diseases from the WTCCC experiment with accumulations of well-imputed rare variants (MAF < 1% and info score of at least 0.4) within genes (as defined by the UCSC human genome database). Each point represents a gene, plotted according to the observed −log_10_ *P*-value of association (*y*-axis) and the physical position of the midpoint of the transcript (*x*-axis), with those achieving genome-wide significance (*P* < 1.7 × 10^−6^) highlighted in red.

Figure 3 presents Manhattan plots to summarise the association of each disease with accumulations of minor alleles at well-imputed rare variants within genes, after correction for the three axes of genetic variation as covariates in the logistic regression model. In these Manhattan plots, each point represents a gene (as defined by the UCSC human genome database), and those achieving genome-wide significance (Bonferroni correction for 30,000 genes, *P* < 1.7 × 10^−6^) are highlighted in red. There was no evidence of residual population structure, not accounted for by the three axes of genetic variation, with genomic control inflation factors [Devlin and Roeder, 1999 less than one for all seven diseases (Supporting Information Figure S3).

**Fig. 4:**
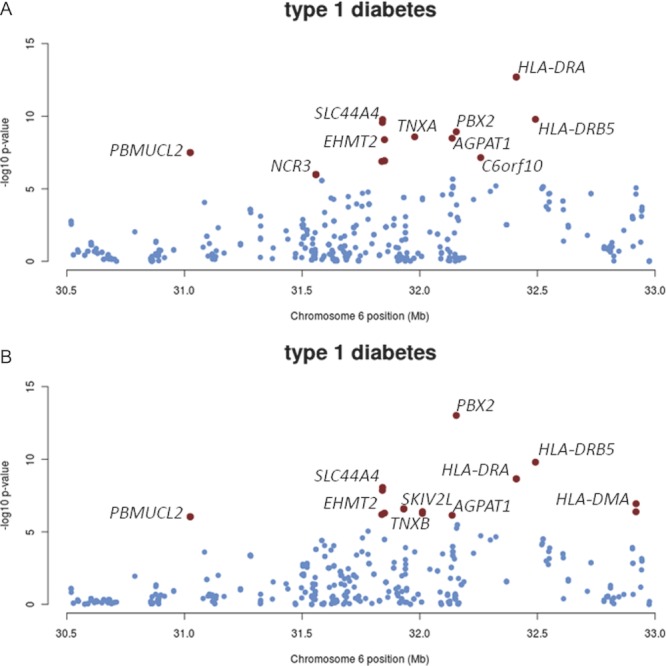
Regional plots summarising association of type 1 diabetes with accumulations of well-imputed rare variants (MAF < 1% and info score of at least 0.4) within MHC genes (as defined by the UCSC human genome database). Each point represents a gene, and those achieving genome-wide significance (*P* < 1.7 × 10^−6^) are highlighted in red. The panels correspond to analyses (A) before and (B) after adjustment for the lead common GWAS SNP (rs9268645) in the region.

We observed genome-wide significant evidence of association of coronary artery disease with rare variants in the gene *PRDM10* (*P* = 4.9 × 10^–8^). The gene contained 122 well-imputed rare variants with mean MAF of 0.23%. Accumulations of minor alleles across these variants were associated with decreased risk of disease: odds ratio 0.828 (0.774–0.886) per minor allele. We also observed 10 genes with genome-wide significant evidence of rare variant association with type 1 diabetes, all located within the major histocompatibility complex (MHC) (Table 1 and Figure 4). The strongest signal of association was observed for *HLA-DRA* (*P* = 2.0 × 10^−13^), which has been previously implicated in susceptibility to type 1 diabetes [Nejentsev et al., 2007. Accumulations of minor alleles at rare variants in nine of the MHC genes were associated with reduced risk of type 1 diabetes (Table 1). The only gene demonstrating evidence of association of accumulations of minor alleles with increased risk of type 1 diabetes was *TNXA*, with odds ratio 2.346 (1.772–3.107) per minor allele.

**Table I:** Genes demonstrating genome-wide significant (*P* < 1.7 × 10^−6^) evidence of rare variant association with type 1 diabetes in the MHC, before and after adjustment for the lead common GWAS SNP (rs9268645) in the region

Gene symbol	Build 37 chromosome 6 position (bp)		Number of rare variants	Mean MAF (%)	Analysis adjusted for three principal components only		Analysis adjusted for three principal components and rs9268645	
	Start	Stop			*P*-value	OR (95% CI)	*P*-value	OR (95% CI)
Genome-wide significant before adjustment for rs9268645								
*HLA-DRA*	32,407,646	32,412,821	23	0.32	2.0 × 10^−13^	0.556 (0.476–0.650)	2.2 × 10^−9^	0.642 (0.555–0.742)
*HLA-DRB5*	32,485,162	32,498,006	43	0.51	1.6 × 10^−10^	0.746 (0.682–0.817)	1.6 × 10^−10^	0.738 (0.673–0.810)
*SLC44A4*	31,837,321	31,846,823	27	0.22	1.7 × 10^−10^	0.556 (0.465–0.666)	8.7 × 10^−9^	0.586 (0.489–0.703)
*PBX2*	32,152,509	32,157,963	13	0.22	1.2 × 10^−9^	0.375 (0.273–0.514)	9.5 × 10^−14^	0.290 (0.210–0.402)
*TNXA*	31,976,196	31,981,050	7	0.41	2.6 × 10^−9^	2.346 (1.772–3.107)	2.4 × 10^−4^	1.719 (1.287–2.295)
*AGPAT1*	32,135,989	32,139,282	5	0.24	3.3 × 10^−9^	0.118 (0.058–0.239)	7.2 × 10^−7^	0.169 (0.084–0.342)
*EHMT2*	31,847,536	31,853,019	16	0.23	4.1 × 10^−9^	0.437 (0.332–0.576)	5.1 × 10^−7^	0.484 (0.365–0.643)
*PBMUCL2*	31,021,983	31,027,653	25	0.35	3.2 × 10^−8^	0.757 (0.686–0.836)	9.1 × 10^−7^	0.777 (0.702–0.859)
*C6orf10*	32,256,302	32,261,812	22	0.41	7.1 × 10^−8^	0.748 (0.673–0.831)	1.9 × 10^−5^	0.793 (0.713–0.882)
*NCR3*	31,557,050	31,560,762	10	0.23	1.0 × 10^−6^	0.436 (0.312–0.608)	1.6 × 10^−4^	0.518 (0.368–0.729)
Genome-wide significant after adjustment for rs9268645								
*HLA-DMA*	32,917,411	32,920,899	13	0.35	8.6 × 10^−6^	0.606 (0.389–0.942)	1.1 × 10^−7^	0.540 (0.430–0.678)
*SKIV2L*	31,926,580	31,937,532	33	0.20	4.0 × 10^−5^	0.706 (0.507–0.984)	2.6 × 10^−7^	0.640 (0.540–0.759)
*TNXB*	32,008,931	32,014,384	10	0.35	4.3 × 10^−5^	0.589 (0.355–0.978)	4.1 × 10^−7^	0.513 (0.396–0.664)

Common SNPs in the MHC have been previously associated with the disease [Barrett et al., 2009; The Wellcome Trust Case Control Consortium, 2007, although fine-mapping of the underlying causal variant(s) has been hindered by the extensive LD across the region. We thus repeated our analyses in this region, testing for association of type 1 diabetes with rare variants within MHC genes after adjustment for the lead GWAS SNP (rs9268645) [Barrett et al., 2009, with genotypes coded by the number of minor alleles, included as an additional covariate in the logistic regression model (Table 1 and Figure 4). The common SNP could not fully explain rare variant associations of type 1 diabetes with any of the MHC genes, but dramatically reduced significance with *TNXA* (*P* = 2.6 × 10^−9^ before adjustment; *P* = 2.4 × 10^−4^ after adjustment). After adjustment, three additional MHC genes achieved genome-wide significant evidence of rare variant association with type 1 diabetes: *HLA-DMA* (*P* = 1.1 × 10^−7^), *SKIV2L* (*P* = 2.6 × 10^−7^) and *TNXB* (*P* = 4.1 × 10^−7^).

## DISCUSSION

GWAS has been extremely successful in identifying genetic loci contributing effects to a wide range of complex human traits [Hindorff et al., 2009 including diseases such as type 2 diabetes [Voight et al., 2010 and Crohn's disease [Franke et al., 2010, and quantitative phenotypes such as body mass index [Speliotes et al., 2010 and height [Lango Allen et al., 2010. However, despite the success of this approach, much of the genetic component of these traits remains, as yet, unexplained [Manolio et al., 2009. Most of the confirmed associations within these loci are with common variants, of modest effect, which well-designed GWAS is adequately powered to detect. It has thus been suggested that much of the ‘missing heritability’ of complex human traits can be attributed to rare genetic variation [Bansal et al., 2010, which is not well captured by GWAS genotyping products. The most comprehensive approach to assaying rare genetic variation is through large-scale next-generation re-sequencing experiments. However, despite advances in the cost-effectiveness of these technologies, whole-genome re-sequencing of the large cohorts of individuals required to detect rare variant association with complex traits, genome-wide, still represents an infeasible financial investment for most research groups.

We demonstrate here, by simulation, that imputation from an existing scaffold of GWAS genotype data using publicly available high-density reference panels, such as those made available through the 1000 Genomes Project [The 1000 Genomes Project Consortium, 2010, has the potential to identify rare variant associations with complex traits, without the need for costly re-sequencing experiments. These results are entirely consistent with other published simulation studies investigating the performance of rare variant association methodology using imputation up to re-sequencing data, either from an external reference panel [Li et al., 2010, or from a subset of the analysis cohort [Zawistowski et al., 2010. Overall, our results suggest that a reference panel of 4,000 individuals offers noticeable gains in power over 500 individuals only when the spectrum of causal variants is loaded with rarer variants (i.e. in our simulations, when the maximum MAF of any individual causal variant is 0.5%, rather than 1%). In this scenario, imputation of the analysis cohort from the GWAS scaffold can achieve much of the power to detect rare variant association obtained by the gold-standard re-sequencing strategy. Our simulations assumed a GWAS scaffold with the same characteristics, in terms of allele frequency profile and density, as the Illumina Human660W-Quad BeadArray. We would expect the quality of imputed rare variants to be improved with more dense GWAS scaffolds, such as the Illumina HumanOmni5-Quad, although this evaluation is beyond the scope of this study.

We have considered a relatively simple underlying model for the association of the trait with multiple rare variants within the gene. More complex models might incorporate selection and/or different directions of effect of the causal variants on the trait. However, at present, we do not fully appreciate the likely effect of rare variants within a gene or pathway on complex human traits, although it is clear that the true underlying association model will impact the power of GRANVIL. Nevertheless, it is less obvious that the underlying association model will impact the relative performance of GRANVIL applied to rare variation derived from imputation as compared to that assayed through re-sequencing.

Our simulation study assumes that the analysis cohort and reference panel are ascertained from the same population, and thus are perfectly matched in terms of their rare variant profile. However, with publicly available reference panels, this is unlikely to be the case. Indeed, the Phase I 1000 Genomes Project reference panel (June 2011 interim release) consists of phased individuals from multiple populations that together incorporate a wide range of ancestry groups [The 1000 Genomes Project Consortium, 2010. One cost-efficient approach to address this issue is to consider re-sequencing a small number of individuals from the analysis cohort to supplement the reference panel. This strategy has been successfully applied in identifying association of a population-specific imputed rare variant with sick sinus syndrome in Iceland [Holm et al., 2011.

Our simulation study also assumes that all rare causal variants in the gene have the same impact on the trait, for example, that they all result in loss-of-function of the gene product. The GRANVIL software makes the same underlying assumption, and hence power to detect rare variant association will be maximised. Under alternative models of rare variant association with the trait, which consider causal variants within the gene to result in both loss- and gain-of-function of the gene product, the power of GRANVIL will be reduced for all design strategies. For these models, powerful methods exist for detecting association with rare genetic variation [Asimit et al., 2012; Neale et al., 2011; Wu et al., 2011. Many of these methods, such as C-alpha [Neale et al., 2011, require direct genotype calls. These approaches could make use of ‘best guess’ genotypes from imputation, although further development is required to appropriately allow for the posterior distribution of calls for imputed variants.

The encouraging results of our simulation study prompted us to re-assess the evidence of rare variant association with seven diseases from the WTCCC experiment [The Wellcome Trust Case Control Consortium, 2007. We were able to recover genotypes at more than 5 million ‘high-quality’ imputed rare variants, even with the Affymetrix GeneChip 500K Mapping Array Set as a scaffold, which would not be expected to capture variation as well as more recent higher density genotyping products. Principal components analysis identified three axes of genetic variation that capture fine-scale population structure within the United Kingdom. After adjustment for these three components as covariates in our logistic regression modelling, there was no discernable residual inflation in rare variant association statistics, indicating that any additional fine-scale population structure had no impact on our analysis. We identified association of coronary artery disease with accumulations of minor alleles at rare variants in *PRDM10* at genome-wide significance. This gene has not been previously implicated in susceptibility to coronary artery disease or related cardio-metabolic phenotypes, and this association signals warrant follow-up in independent cohorts, either through genotyping of rare variants in the gene or re-sequencing. We also identified genome-wide significant evidence of association of type 1 diabetes with accumulations of minor alleles at rare variants in multiple genes from the MHC. This region has been previously associated with type 1 diabetes, both through common variant GWAS and analysis of classical human leukocyte antigen (HLA) haplotypes. Further work is required to dissect the complex genetic contribution of common and rare variation in this region to susceptibility to type 1 diabetes and other autoimmune disorders.

The results of our analyses presented here have major implications for the design and analysis of genome-wide rare variant association studies of complex human traits. Our results clearly highlight the potential for the detection of rare variant associations by using existing GWAS genotype data, supplemented with imputation from publicly available high-density reference panels, without the need for costly whole-genome re-sequencing experiments. Although imputation can never replace the gold-standard approach of whole-genome re-sequencing, it provides a powerful, cost-effective alternative that only requires a scaffold of GWAS genotype data, which may already be available. This message will bring encouragement to research groups who do not have sufficient funding to consider whole-genome, or even whole-exome sequencing as a financially viable approach to assaying rare genetic variation. It is clear that GWAS still have the potential to offer an exciting opportunity for gene discovery, conceivably leading to substantial advancements in our understanding of the genetic architecture underlying complex human traits.

## Appendix

### CALCULATION OF THE RESIDUAL TRAIT VARIANCE

Determine the number of minor alleles carried by the *i*th individual in the analysis cohort across all causal variants in the 50 kb gene, denoted *m_i_*. The expected trait value for the *i*th individual, denoted by *μ_i_*, takes the value 1 if *m_i_* > 0, and 0 otherwise. Assuming an analysis cohort of *N* individuals, the mean expected trait value is given by

with corresponding genetic variance given by

Assuming that the rare causal variants explain 100*λ*% of the overall trait variance, it follows that the residual variance is given by

## Notes

Disclaimer: Supplementary materials have been peer-reviewed but not copyedited.

**Fig. 1.** Number of rare variants observed (or well imputed) for different strategies for assaying genetic variation in a 50 kb gene, as a function of the size of the reference panel. **Fig. 2.** Power, at a nominal significance level of *P* < 0.05, to detect association of an accumulation of minor alleles with a quantitative trait, for different strategies for assaying rare genetic variation in a 50 kb gene, as a function of the genotyping and sequencing error rate. **Fig. 3.** Quantile-quantile plots summarising association of seven diseases from the WTCCC experiment with accumulations of well imputed rare variants (MAF < 1% and info score of at least 0.4) within genes (as defined by the UCSC human genome database).
