# Supplementary material for: Genome-Wide Association Analysis of Imputed Rare Variants: Application to Seven Common Complex Diseases
Source: Genet Epidemiol. 2012 Sep 5;36(8):785–96. doi: 10.1002/gepi.21675 (PMC3569874; doi:10.1002/gepi.21675)

Supplementary Figure Legends

Supplementary Figure 1. Number of rare variants observed (or well imputed) for different strategies for assaying genetic variation in a 50kb gene, as a function of the size of the reference panel. Multiple causal variants in the gene contribute jointly to 5% of the overall trait variation. The panels correspond to two specific trait association models: (a) the maximum MAF of any individual causal variant is 1%, and the total MAF of all causal variants is 5%; and (b) the maximum MAF of any individual causal variant is 0.5%, and the total MAF of all causal variants is 2%.

Supplementary Figure 2. Power, at a nominal significance level of $p<0.05$, to detect association of an accumulation of minor alleles with a quantitative trait, for different strategies for assaying rare genetic variation in a 50kb gene, as a function of the genotyping and sequencing error rate. Multiple causal variants in the gene contribute jointly to 5% of the overall trait variation. The maximum MAF of any individual causal variant is 1%, and the total MAF of all causal variants is 5%. The size of the reference panel for imputation is 120 individuals. The panels correspond to: (a) no missing genotype data; and (b) 1% missing genotype data at random.

Supplementary Figure 3. Quantile-quantile plots summarising association of seven diseases from the WTCCC experiment with accumulations of well imputed rare variants (MAF<1% and info score of at least 0.4) within genes (as defined by the UCSC human genome database). Each point represents a gene, plotted according to the observed $-\log_{10}$ *p*-value of association (*y*-axis) and that expected under the null hypothesis (*x*-axis).

Supplementary Figure 1

(a)

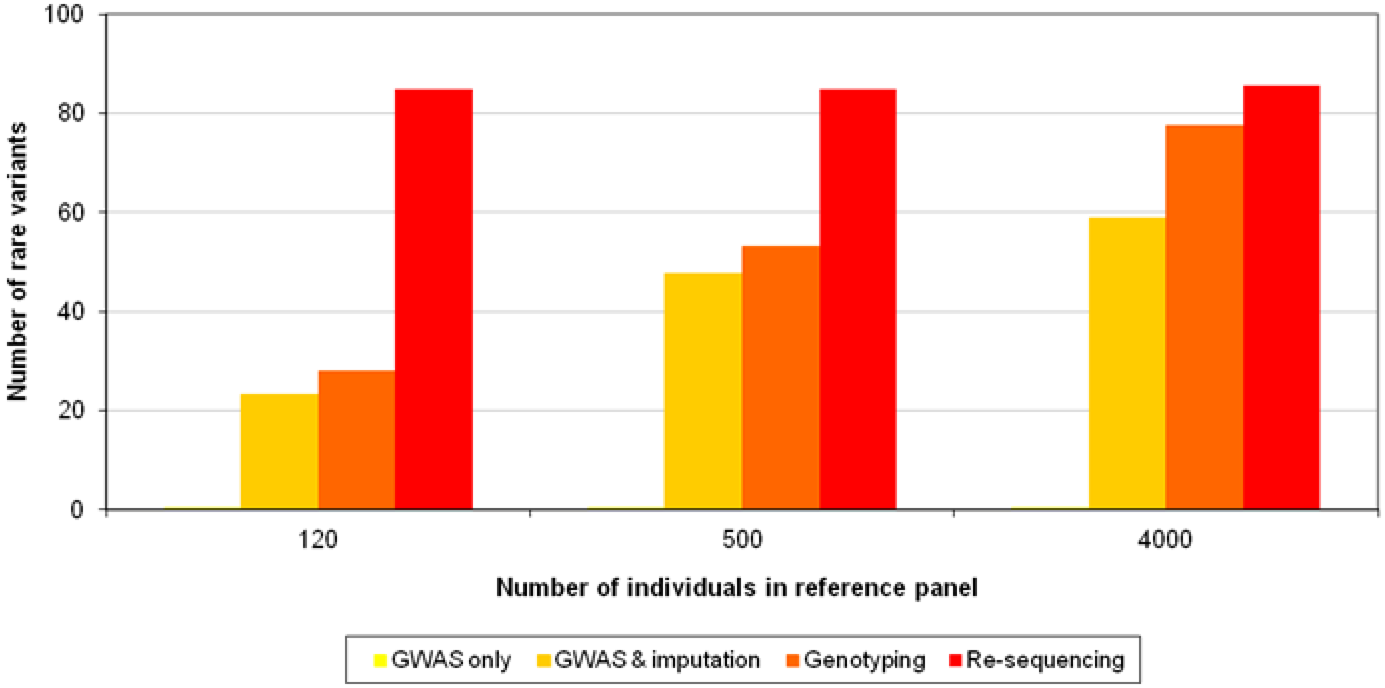

(b)

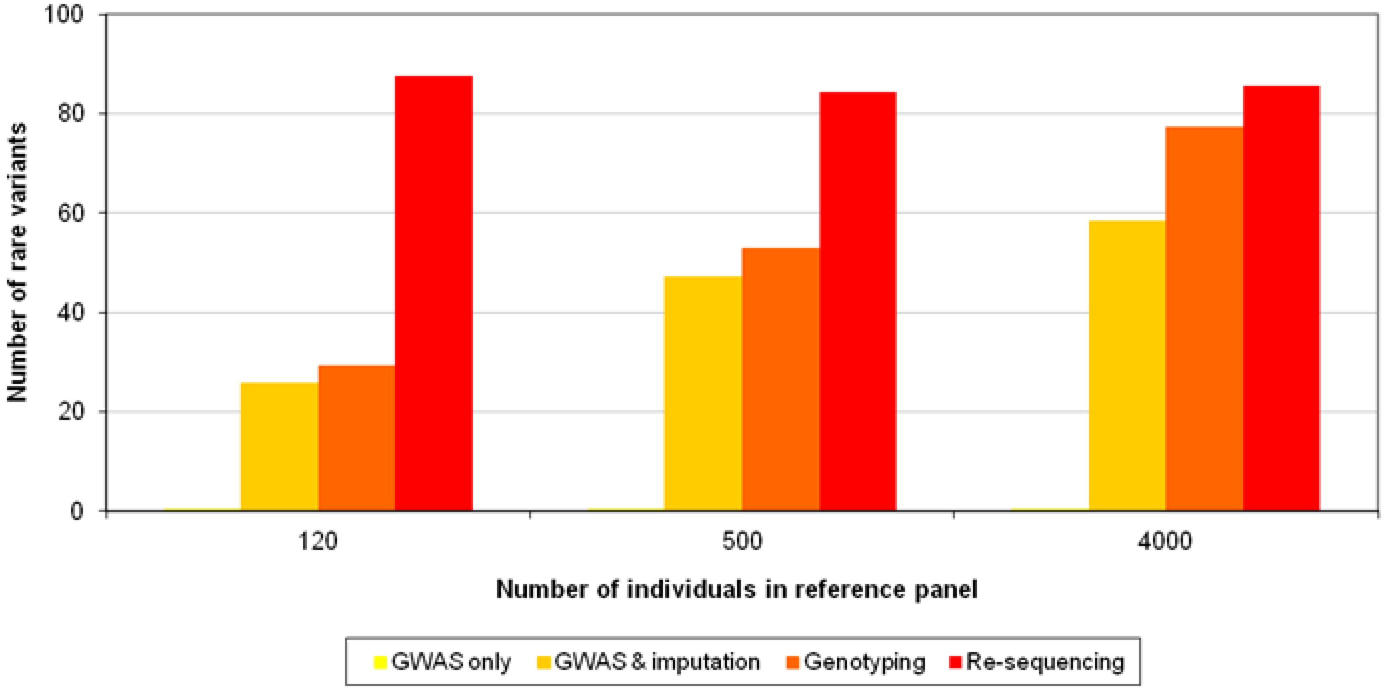

Supplementary Figure 2

(a)

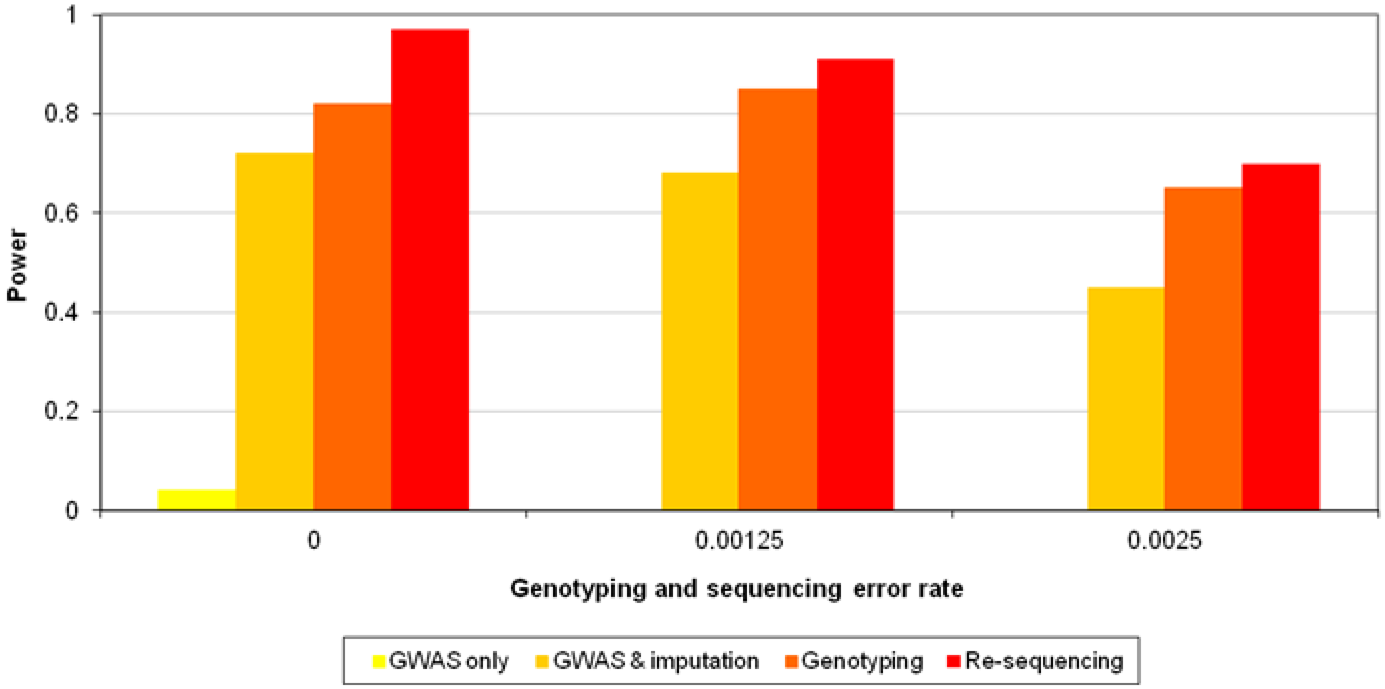

(b)

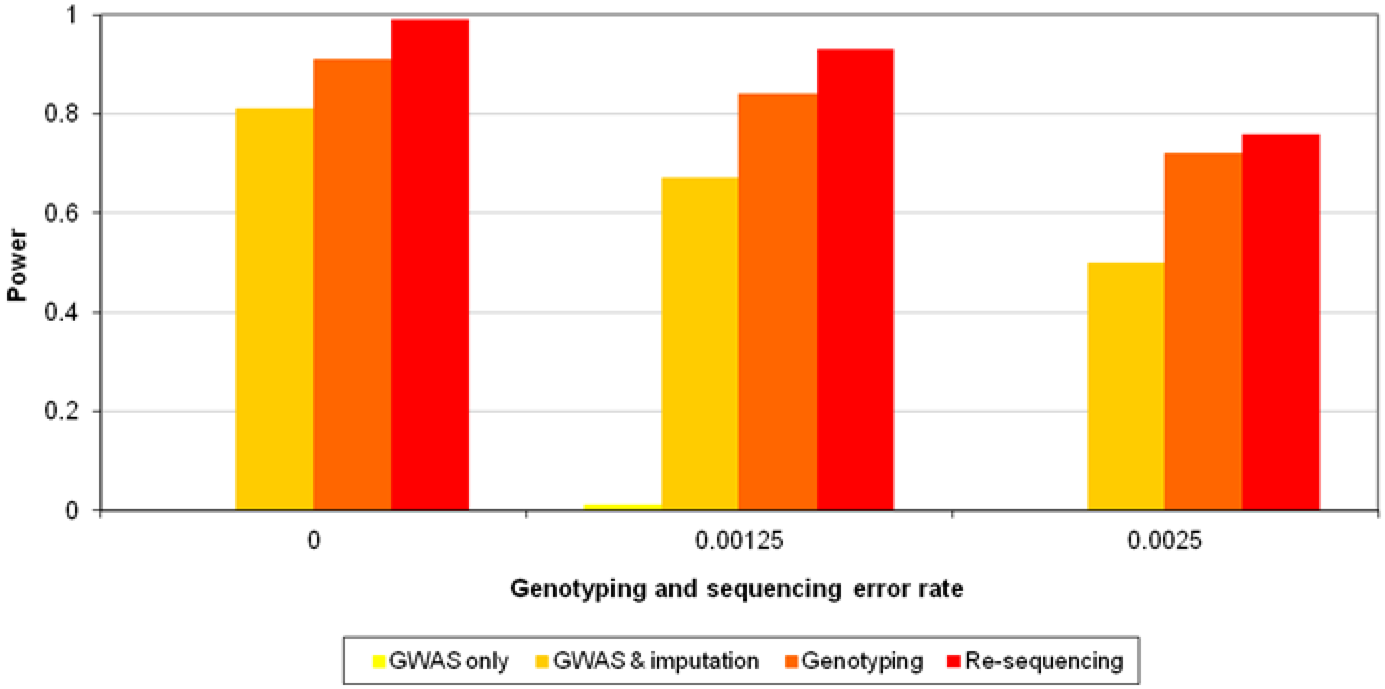

Supplementary Figure 3

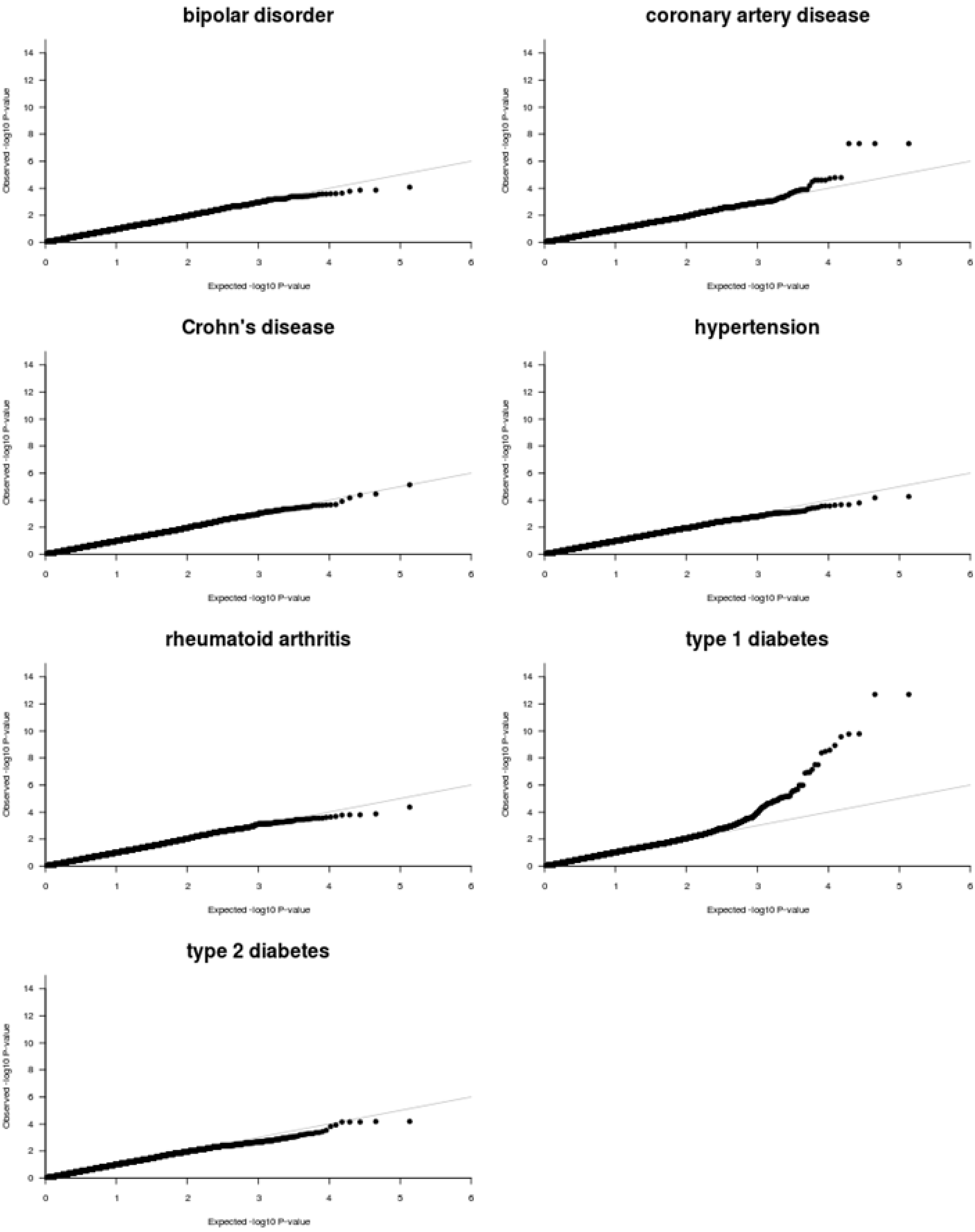

Supplement: Supplementary file 1 — Fig. 1. Number of rare variants observed (or well imputed) for different strategies for assaying genetic variation in a 50 kb gene, as a function of the size of the reference panel. Fig. 2. Power, at a nominal significance level of P < 0.05, to detect association of an accumulation of minor alleles with a quantitative trait, for different strategies for assaying rare genetic variation in a 50 kb gene, as a function of the genotyping and sequencing error rate. Fig. 3. Quantile-quantile plots summarising association of seven diseases from the WTCCC experiment with accumulations of well imputed rare variants (MAF < 1% and info score of at least 0.4) within genes (as defined by the UCSC human genome database). [file gepi0036-0785-SD1.pdf]
